# Proline-Directed Androgen Receptor Phosphorylation

**DOI:** 10.4172/1747-0862.1000075

**Published:** 2013-08-26

**Authors:** Yanfei Gao, Shaoyong Chen

**Affiliations:** Division of Hematology/Oncology, Beth Israel Deaconess Medical Center, Harvard Medical School 330 Brookline, MA 02115, USA

**Keywords:** Androgen receptor, Phosphorylation, Proline-directed serine/threonine phosphorylation, Kinases, Phosphatase

## Abstract

The androgen receptor (AR) has been identified for decades and mediates essential steroid functions. Like most of biological molecules, AR functional activities are modulated by post-translational modifications. This review is focused on the reported activities and significance of AR phosphorylation, with particular emphasis on proline-directed serine/threonine phosphorylation that occurs predominantly on the receptor. The marked enrichment of AR phosphorylation in the most diverse N-terminal domain suggests that targeting AR phosphorylation can be synergistic to antagonizing the C-terminal domain by clinical antiandrogens.

## Introduction

### Androgen receptor (AR)

The identification of steroid receptors (including AR) in the mid-1980s led to the definition of a family of ligand-mediated transcription factors that occupy specific chromatic locus for gene regulation. The next chapter in advancements is the identification of cofactors participating chromatin remodeling, including histone “writers”, “erasers”, and “readers” that have continuously been researched up to now. Besides the well-established AR activity in mediating transcriptional activation, recent studies further revealed novel functions of AR in transcriptional repression, genomic translocation, and mTOR activation [1–6]. Abnormal AR activity is associated with various pathogeneses such as male infertility, androgen-insensitivity syndrome (AIS), polycystic ovarian syndrome (PCOS), spinal and bulbar muscular atrophy (SBMA), rheumatoid arthritis, hirsutism, baldness, acne, breast cancer, and prostate cancer (PCa).

**Figure 1:**
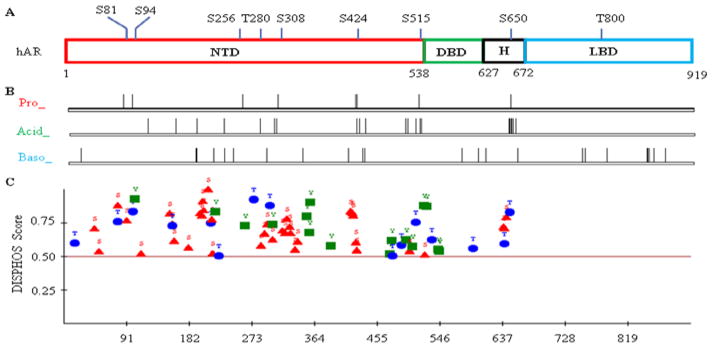
Theoretical and computational characterization of human androgen receptor (hAR) phosphorylation.(A) hAR amino acid linear organization indicated of structural domains and proline(Pro)-directed phosphorylable residues (GenBank: M20132.1); (B) Motif Scan Graphic analysis ofhAR subjected to Pro-directed Ser/Pro kinase (Pro_ST_Kin or Pro_), Acid Ser/Pro kinase (Acid_ST_Kin or Acid_); and Baso Ser/Pro kinase (Baso_ST_Kin, or Baso_) (http://scansite.mit.edu); (C) Phosphorylation analysis of hAR by the DISorder-enhanced PHOSphorylation predictor (DISPHOS, http://www.ist.temple.edu/DISPHOS) program.

The AR molecule is structurally organized into distinct domains: the N-terminal domain (NTD) that has a potent activation function 1 (AF-1), DNA-binding domain (DBD), hinge domain (H), and ligand-binding domain (LBD) that binds to androgens and has a modest activation function 2 (AF-2) (Figure 1A). The AR protein shares highly structural similarities in the DBD and LBD with other steroid nuclear receptor family members, such as glucocorticoids receptor (GR), mineral corticoid receptor (MR), and progesterone receptor (PR). However, its NTD and hinge regions are unique and structurally disordered, and share marked diversity among family members. The NTD and hinge domain are also less conservative than the DBD and LBD among ARs from different species. Functionally, these distinct domains confer activities such as N-to-C interactions, DNA loading, antiparallel dimerization, and recruitment of cofactors. The AR proteins are also subjected to multiple post-translational modifications such as acetylation, methylation, ubiquitination and sumoylation. This review will be focused on AR phosphorylation, with emphasis on proline (Pro)-directed phosphorylation.

### AR phosphorylation

The identification of AR was immediately followed by the recognition that the receptor is a phosphoprotein and that phosphorylated AR is localized to the nucleus upon ligand stimulation [7–10]. More extensive studies indicated that AR is synthesized as a single 110 kDa protein that is rapidly converted into a 112 kDa phosphoprotein in the absence of hormone, with constitutive phosphorylation at two Pro-directed serines (Ser650 and Ser94); and that androgens can further induce the expression of a 114 kDa isoform which is phosphorylated at additional residues and associated with AR nuclear activities [11–13]. The distribution of these three isoforms can be attributed to the NTD, in particular the length of the outstanding polyglutamine (poly-Q) stretch and the phosphorylation at two adjacent Pro-directed serines (Ser81 and Ser94) [14].

As shown in table 1, AR has more than 150 theoretically phosphorylable residues, which are modestly enriched in the hinge and NTD regions. Interestingly, the AR molecular evolution is associated with a decrease in the serines, an increase in the threonines, and a basically unchanged number of tyrosines. Furthermore, the Pro-directed Ser (7) and Thr (2) are well conserved among species (Table 1). In addition, the human AR also has abundant glycine (G, 97), proline (P, 74), and glutamine (Q, 69). Computational calculation indicates that the Pro-directed Ser residues are subjected to phosphorylation by Pro-directed Ser/Pro kinase (Figure 1B). While the Acid Ser/Pro kinase theoretically covers AR but the C-terminal LBD, the Baso Ser/Pro kinase can potentially phosphorylate all four domains (Figure 1B). This result is in concordance with an analysis by a distinct program, indicating that the N-terminal region (NTD-DBD-H; in particular the NTD) is the major phosphorylation locus of AR (Figure 1C). Consistently, multiple studies indicated that the NTD is the predominant AR phosphorylation region and the phosphorylation occurs mainly on Ser and Thr residues [15–17]. Lower phosphorylation frequency identified in DBD and especially the LBD is rather due to their highly structural conformation than low percentage of phosphorylable residues, considering that phosphorylation occurs mainly on intrinsically unstructured locus (Figure 1C and Table 1) [18].

The enrichment of phosphorylation at NTD implies its function in AR-mediated transactivation. Indeed, AR phosphorylation status is strongly correlated with the transcriptional function and the agonistic activity of ligands [19]. Consistently, the NTD (the major AR phosphorylation region) bears AR activation mediated by the HER2/EGFR and IL-6/ MAPK pathways [20,21]; and Aurora-A and Ack1 also activate AR by phosphorylating the NTD (Thr282/Ser293 versus Tyr267/Tyr363, respectively) [22,23]. Furthermore, AR phosphorylation is involved in AR degradation by the proteasome-dependent pathway: while phosphorylation at Ser578 promotes AR-Mdm2 (E3 ligase murine double minute-2) association and AR degradation, phosphorylation on Tyr (534) attenuates AR ubiquitination and interaction with the E3 ligase CHIP (COOH terminus of Hsp70-interacting) protein, leading to increased AR expression [24–26]. In addition, AR phosphorylation is also linked to additional modifications such as AR acetylation [27].

### Proline-directed AR phosphorylation

Despites the scattered reports on AR phosphorylation at various residues, it is well documented that the receptor phosphorylation occurs predominantly on the Pro-directed serines, as evidenced by the studies based on phosphoamino acid and mass-spec analyses (data not shown) [28]. As indicated in figure 1A and table 1, AR totally has seven Pro-directed serines, with six located at the NTD and one at the hinge region. Functionally, AR phosphorylation at Ser308 by cyclin D3/CDK11p58 reduced transcriptional activity [29], while the functional significance of S515 phosphorylation appears different between exogenous and endogenous studies [13,30,31]. In addition, S424 and S515 phosphorylation contributes to AR nuclear localization and functions against receptor aggregation upon hormone treatment [32].

**Figure 2:**
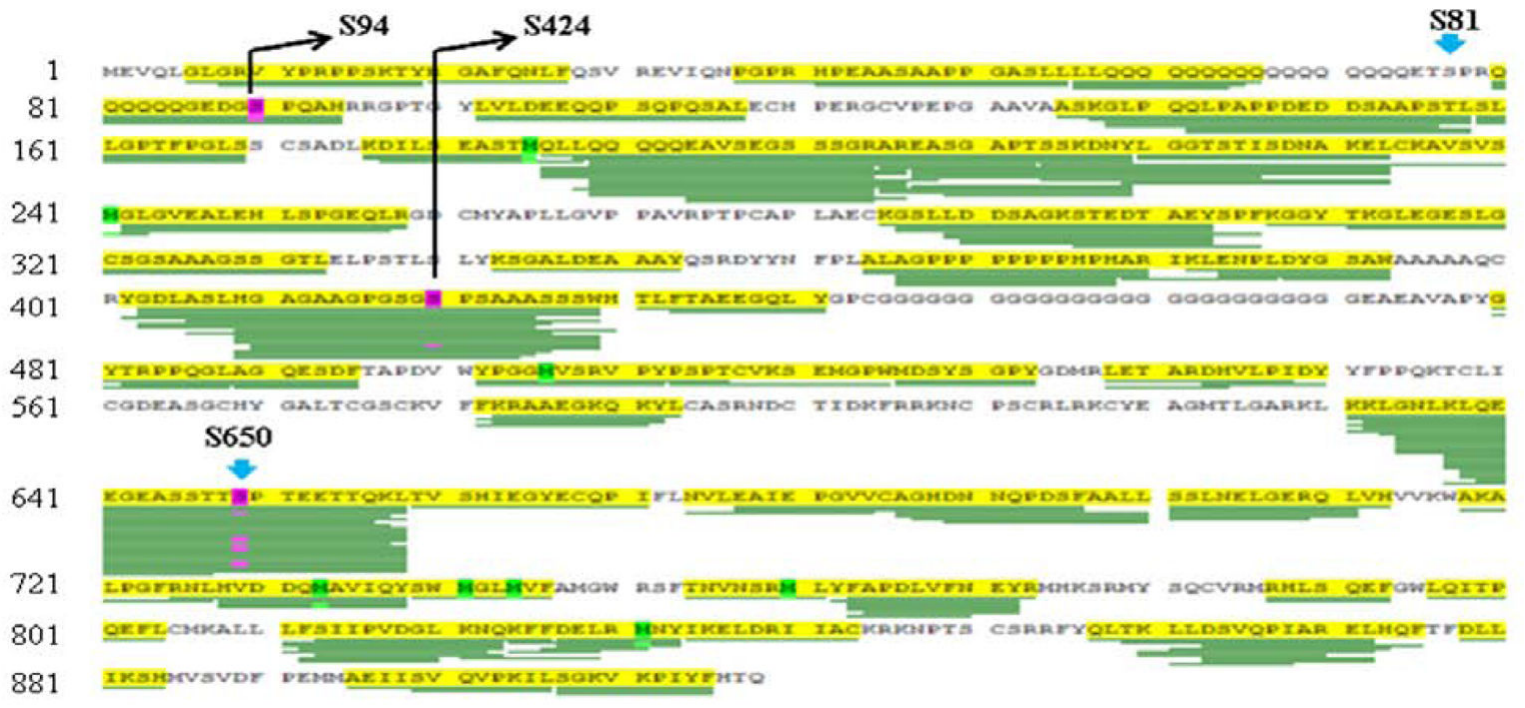
A typical mass-spec analysis in AR study. LNCaP cells in androgen-deprived medium were treated with DHT (dihydrotestosterone) and AR was harvested by Co-IP (Co-immunoprecipitation) for mass-spec analysis that was aligned to human AR (GenBank: M23263.1). Highlighted are identified phosphorylation corresponding to Ser94 and Ser650(GenBank: M20132.1), with Ser424 at lower frequency. Phosphorylation on Ser81 is not identified, likely due to its particular location that affects fragmentation during processing.

**Figure 3:**
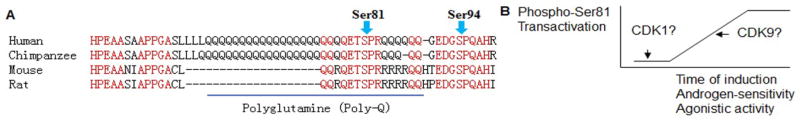
AR linear amino acid sequences in the vicinity of Ser81 and Ser94 residues.(A) Alignment of human, chimpanzee, mouse and rat AR with highlighted conserved residues (in red) and polyglutamine region (underlined); (B) Schematic drawing indicates that Ser81 phosphorylation is correlated to AR functional activities.

Next, we will concentrate on three Pro-directed Ser residues (Ser81, Ser94, and Ser650) that are most robustly phosphorylated based on multiple phosphoamino acid and mass-specanalyses (Figure 2) [12,14]. Although Ser81 is apparently the highest androgen-stimulated AR phosphorylation residues, this event cannot be readily captured by mass-spec due to its particular embedment in the Poly-Q stretch that would compromise the fragmentation efficiency during digestion and processing (Figure 2 and 3) [17].

### Pro-directed Ser81 phosphorylation

Ser81 is the most stoichiometrically serine residue phosphorylated in response to androgens and its phosphorylation occurs with distinct dynamics compared to other AR phosphorylation residues [12,17]. The particular activities of Ser81 phosphorylation can be attributed to its extraordinary positioning in an unusual polyglutamine (poly-Q) stretch in the NTD (Figure 3A). Interestingly, a linear increase in the length of poly-Q is proportional to the time of animal divergence, suggesting an association of polyglutamine expansion with evolution of the higher primate species [33]. Pathologically, the polymorphic poly-Q is causative to certain neurodegenerative diseases, as exemplified by the neuromuscular disorder SBMA [34]. In the molecular settings, expansion of the poly-Q track led to abnormal AR protein folding, aggregation, and interaction with other proteins, resulting in excessive AR degradation and compromised AR transcriptional capacity [35].

In the prostate cancer (PCa), Ser81 phosphorylation contributes to cell growth, AR-mediated transcription, and AR sensitivity to ligand [36–39]. Although transient transfection assay yielded little effect of Ser81 phosphorylation on AR-mediated transcription [17,36], studies based on PCa and endogenous genes indicated that this phosphorylation had pronounced effects on AR nuclear distribution, chromatin binding, and transactivation functions [37,39,40]. Consistently, attenuation of AR Ser81 phosphorylation by antagonists for CDK1, CDK9, TOPO1 (topoisomerase I) and HER2 led to decreased PCa cell growth and AR nuclear functions such as chromatin binding and transcriptional activation [37,39,41,42]. Interestingly, AR Ser81 phosphorylation can also function in the PCa epithelial-stromal interactions, mediated by the ERK pathway that may directly phosphorylate this residue [36,43].

Mechanistically, the initial work from our Lab identified Cdk1 as a Ser81 kinase that can phosphorylate Ser81 to stimulate AR nuclear functions [36]. Further studies indicated that CDK9 specifically phosphorylate AR at Ser81 upon androgen stimulation, leading to productive AR chromatin binding for sustained transcription [37,39]. Consistently, DNA binding has been implicated in Ser81 phosphorylation and androgens-induced AR localization to the active chromatin may be associated with phosphorylation by specific protein kinase occupying the locus [14,44,45]. Together, these findings suggested that CDK1-mediated Ser81 phosphorylation may account for the basal Ser81 phosphorylation that can initiate AR loading to the chromatin locus, followed by CDK9-mediated phosphorylation that is coupled to transcriptional activation (Figure 3B). At molecular levels, Ser81 phosphorylation is implicated in AR nuclear distribution and its interaction with co-factors, such as CBP (CREB binding protein) and GRIP1 (glucocorticoid receptor-interacting protein 1) [45]. In addition, a recent report also indicated that Ser81 phosphorylation mediates the interaction between AR and Pin1, a peptidyl-prolyl cis-trans isomerase (PPIase) that specifically isomerizes Pro-directed phospho-Ser/Thr motifs [46].

### Pro-directed Ser94 phosphorylation

The Ser94 locates to the C-terminal of the NTD poly-Q track (Figure 3A) and its phosphorylation can occur in the absence of ligands [12]. Ser94 together with Ser81 and Ser650 are the three Pro-directed residues that are most substantially phosphorylated AR residues (Figure 2) [17]. The functional significance of Ser94 phosphorylation is unknown; although a bias was found for Ser94 phosphorylated AR distribution in the cytoplasm in the absence of androgens [40]. In addition, increased Poly-Q length is associated with enhanced Ser94 phosphorylation while transient transfection study has indicated that Ser94 phosphorylation has minimal effect on AR-mediated activation of exogenous promoters [13,17,33]. The Ser94 kinase(s) are also unknown, although CDK1 and CDK5 but not CDK9 can be the candidates [36,37]. Considering Ser94 phosphorylation can happen in the absence of androgens, it remains to be determined whether this phosphorylation is involved in the interaction between AR with the HSP90 complex in the cytoplasm that binds to and stabilizes the new-synthesized AR proteins [47].

### Pro-directed Ser650 phosphorylation

**Figure 4:**
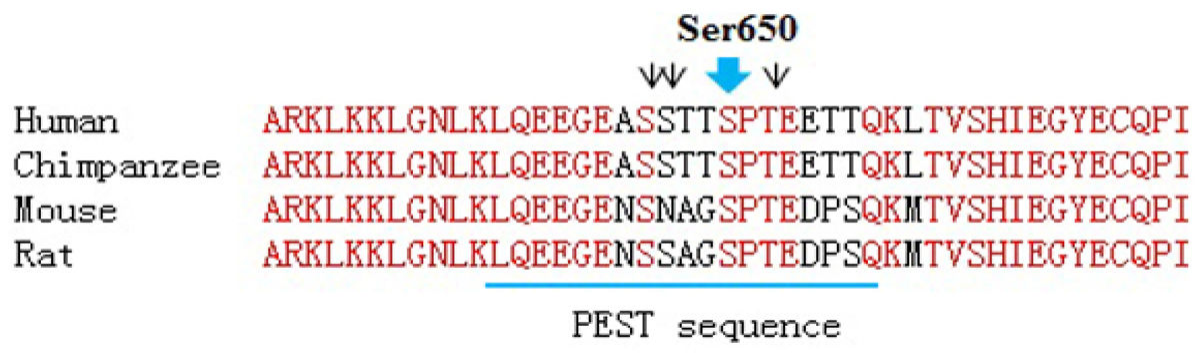
AR linear amino acid sequences of the hinge domain. Alignment of human, chimpanzee, mouse and rat AR with highlighted conserved residues (in red) and PEST sequences (underlined). Black arrow heads indicated additional phosphorylation residues (Ser646, Ser647, and Thr652) identified in the hinge region by our mass-spec studies (data not shown).

The Ser650 in the only Pro-directed serine residue that locates outside of the NTD. It resides in the hinge region and centers on the diverse PEST sequence that potentially mediates AR protein degradation (Figure 4). The hinge region contains part of the NLS (nuclear translocation signal) and regulates AR transactivation and nuclear localization, and is one major target site for modifications (acetylation, ubiquitination and methylation) [48,49]. The hinge region is also enriched in phosphorylable residues and indeed, mass-spec analysis has identified phosphorylation occurring at Ser650 and several adjacent residues (Table 1, Figure 4; data not shown). Although transient transfection assays suggested thatS650 phosphorylation has no or minimal effects on AR functional activities, study based on endogenous AR indicated that the stress kinases (JNK (c-Jun N-terminal kinase) and p38) regulate Ser 650 phosphorylation and AR nuclear export [13,17,28]. The functions of phosphorylation at the adjacent residues (Ser646, Ser647, and Thr652) are unknown (Figure 4).

### Phosphoprotein phosphatases targeting the Pro-directed Ser/Thr residues of AR

The findings that phosphorylated AR is transcriptionally active are essentially consistent with the observations that AR dephosphorylation impairs receptor functional nuclear activities like ligand binding [50,51]. Consistently, the tumor antigens simian virus 40 small t antigen (ST) can mediate PP2A (phosphoprotein phosphatase 2A) binding to AR, leading to AR dephosphorylation at five Pro-directed phosphoserines in the NTD and reduction in AR activities [51]. Significantly, PP2A activity is attenuated in the androgen-independent C4-2 PCa cells as compared with the parental androgen-dependent LNCaP cells [52]. Furthermore, PPP2R2C (a PP2A regulatory subunit) was down-regulated in advanced PCa to drive castration-resistance [53]. In addition, study from our Lab indicated PP1 (phosphoprotein phosphatase 1) stimulates AR nuclear functions (in opposite to that of the PP2A), mediated by PP1-elicited dephosphorylation of Ser650 in the hinge region [54]. These findings are fundamentally in line with the report that caveolin-1 can increase nuclear functions of the phosphorylated AR by binding to and inhibiting the PP1 and PP2A [55].

### Clinical implications of AR phosphorylation

AR phosphorylation has been extensively implicated in pathogeneses, as exemplified by that the development of castration-resistant prostate cancer (CRPC) can be attributed to AR phosphorylation at Tyr267 (by the Ack1 pathway) and Ser515/Ser578 (by the EGFR/MAPK signaling), respectively [56,57]. The enrichment of AR phosphorylation at the NTD indicates one therapeutic strategy is to co-target AR phosphorylation and ligand binding functions. Indeed, inhibition of phospho-Ser81 can synergize with anti-androgen to disturb CRPC [36,42]. Significantly, a recent systematic study based on screening 673 human kinases in PCa cells identified six potential targeting kinases (MAP3K11, DGKD, ICK, CIT, GALK2, and PSKH1), and it is important to assess efficacy of antagonizing these candidates in combination with clinical antiandrogens [58]. In addition, as one frontier in AR research, the receptor phosphorylation has emerged as a potential biomarker in clinical analysis. Indeed, breast and prostate cancer studies have been reported based on immunohistochemistry (IHC) assays with the phospho-Ser213, Ser515, and Ser650 antibodies [31,59–62]. These studies can be further substantiated by sufficient antibody validation, such as dose optimization and specific peptide competition analysis.

## Conclusions

In summary, altered and amplified phosphorylation can contribute to abnormal AR activities, including its ligand-independent activation in diseases including PCa. The AR NTD is bestowed with highly selectivity and enriched phosphorylation, providing ample opportunities for specific interventions. Targeting NTD phosphorylation (by kinase and phosphatase modulators) can be applied in synergy with the LBD antagonists (such as antiandrogens) in therapy. Targeting AR phosphorylation is also an option to overcome the AR splicing variants that lose the functional ligand binding capacity and are overexpressed in advanced PCa [63]. Finally, although AR phosphorylation has been extensively studied in receptor activation, its intrinsic connections need to be clarified to AR-mediated transrepression and AR non-genomic functions (like mTOR activation).

## Figures and Tables

**Table 1:** Characterization of AR amino acid composition regarding theoretically phosphorylable residues.

	Total AA	Serine	Theonine	Tyrosine	S/T/Y	Percentage	Pro-Ser	Pro-Thr

Human AR	919	81	37	33	151	%16.43	7	2
Chimpanzee AR	911	81	37	33	151	%16.58	7	2
Mouse AR	899	87	35	34	156	%17.35	7	2
Rat AR	902	92	33	33	158	%17.52	7	2

Human AR NTD	537	56	20	20	96	%17.88	6	1
Human AR DBD	90	4	5	5	14	%15.56	0	0
Human AR Hinge domain	45	4	6	1	11	%24.44	1	0
Human AR LBD	247	17	6	7	30	%12.15	0	1

GenBank entry: human AR (M20132.1); chimpanzee AR (NM_001009012.1); mouse AR (NM_013476.3); and rat AR (NM_012502.1). AA: amino acid; S: Serine/Ser; T: Threonine/Thr; Y: Tyrosine/Tyr.

## Abbreviations

AF-1/2: AR Activation Function 1/2
AIS: Androgen-Insensitivity Syndrome
AR: Androgen Receptor
CBP: CREB Binding Protein
CHIP: E3 ligase COOH Terminus of Hsp70-interacting Protein
Co-IP: Co-immunoprecipitation
CRPC: Castration-resistant Prostate Cancer
DBD: AR DNA-binding Domain
DHT: Dihydrotestosterone
GR: Glucocorticoid Receptor
GRIP1: Glucocorticoid Receptor-Interacting Protein 1
H: AR Hinge Domain
IHC: Immunohistochemistry
JNK: c-Jun N-terminal Kinase
LBD: AR Ligand-Binding Domain
Mdm2: E3 Ligase Murine Double Minute-2
MR: Mineralocorticoid Receptor
NLS: Nuclear Translocation Signal
NTD: AR N-terminal Domain
PCa: Prostate Cancer
PCOS: Polycystic Ovarian Syndrome
PEST Sequence: A Peptide Sequence which is Rich in Proline (P), Glutamic Acid (E), Serine (S), and Threonine (T)
Pin1: Peptidyl-Prolyl Cis-Trans Isomerase (PPIase) 1
Poly-Q: Polyglutamine
PP1/2A: Phosphoprotein Phosphatase 1/2A
PR: Progesterone Receptor
SBMA: Spinal And Bulbar Muscular Atrophy
ST: Tumor Antigens Simian Virus 40 Small T Antigen
